# Lateralization of facial emotion processing and facial paresis in Vestibular Schwannoma patients

**DOI:** 10.1002/brb3.1644

**Published:** 2020-05-12

**Authors:** Stephanie S. A. H. Blom, Henk Aarts, Henricus P. M. Kunst, Capi C. Wever, Gün R. Semin

**Affiliations:** ^1^ Department of Psychology Utrecht University Utrecht The Netherlands; ^2^ Department of Otolaryngology Radboud Institute for Health Sciences Radboud University Medical Center Nijmegen The Netherlands; ^3^ Department of Otolaryngology Maastricht UMC+ Maastricht The Netherlands; ^4^ Department of Otolaryngology – Head & Neck Surgery Leiden University Medical Center Leiden The Netherlands; ^5^ William James Center for Research ISPA – Instituto Universitário Lisboa Portugal

**Keywords:** emotion expression, facial mimicry, facial paresis, hemispheric processing, Vestibular Schwannoma

## Abstract

**Objective:**

This study investigates whether there exist differences in lateralization of facial emotion processing in patients suffering from Vestibular Schwannoma (VS) based on the presence of a facial paresis and their degree of facial functioning as measured by the House Brackmann Grading scale (HBG).

**Methods:**

Forty‐four VS patients, half of them with a facial paresis and half of them without a facial paresis, rated how emotive they considered images of faces showing emotion in the left versus right visual field. Stimuli consisted of faces with a neutral half and an emotional (happy or angry) half. The study had a mixed design with emotional expression (happy vs. angry) and emotional half (left vs. right visual field) of the faces as repeated measures, and facial paresis (present vs. absent) and HBG as between subjects’ factors. The visual field bias was the main dependent variable.

**Results:**

In line with typical findings in the normal population, a left visual field bias showed in the current sample: patients judged emotional expressions shown in the left visual field as more emotive than those shown in the right visual field. No differences in visual field bias showed based on the presence of a facial paresis nor based on patients’ HBG.

**Conclusion:**

VS patients show a left visual field bias when processing facial emotion. No differences in lateralization showed based on the presence of a facial paresis or on patients’ HBG. Based on this study, facial paresis thus does not affect the lateralization of facial emotion processing in patients with VS.

## Graphical abstract

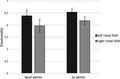

## 1 INTRODUCTION

Recognizing emotions and being able to simulate them—a process generally referred to as facial mimicry—are important facets of human social functioning. These elements are vital in human life. Newborn infants already show a preference for faces and face‐like stimuli (Johnson, 2005), and facial mimicry is considered to be an automatic process (Dimberg, Thunberg, & Grunedal, 2002) that supports a quick understanding of the emotionality of “the other” in social interaction (Niedenthal, 2007). Thus, simulation and mimicry of facial emotion expressions are a human fundamental ability that plays a key role in attending to and interpreting other's facial expressions in human interaction and communication.

However, not all people are blessed with such ability. There are patients who can be assumed to encounter limitations in simulating facial expressions, due to impaired facial functioning such as facial paresis. In line with this idea, there is compelling evidence that impaired facial functioning undermines social functioning, emotional life, and mental health (Cross, Sheard, Garrud, Nikolopoulos, & O'Donoghue, 2000; Fu, Bundy, & Sadiq, 2011; Guntinas‐Lichius, Straesser, & Streppel, 2007; Ishii et al., 2011; Nellis et al., 2017; Ryzenman, Pensak, & Tew, 2005). Because of the association between facial dysfunction and social and emotional factors of quality of life, it is especially relevant to understand whether facial dysfunction in patients impacts specific aspects of facial emotion processing. This study was designed to address this issue and to provide a first test to explore whether facial emotion processing might be impaired in specific patient group that suffers from facial dysfunction—patients with a Vestibular Schwannoma.

Vestibular Schwannoma (VS) refers to a unilateral brain tumor also referred to as an acoustic neuroma (Weinberger & Terris, 2015). Typical clinical symptoms are hearing loss on the affected side, tinnitus, as well as disequilibrium (Weinberger & Terris, 2015). Because a VS is located near the facial nerve, surgical removal of it can cause a degree of unilateral paresis in the patient. To examine the potential disturbing impact of VS on facial emotion processing, we used a well‐documented method that tests a specific facet of facial emotion perception, namely hemispheric lateralization of facial emotion processing. Theories regarding hemispheric lateralization of facial emotion processing generally consider two main viewpoints. Whereas the right hemisphere hypothesis states that all emotions are, generally, processed in the right hemisphere, the valence hypothesis states that the left hemisphere is dominant in processing positive emotions, and that the right hemisphere is dominant in processing negative emotions (e.g., Bourne, 2010).

Support for both viewpoints exist. For instance, right—compared to left—hemispheric processing of facial emotional expressions has often been reported to relate to better discrimination, recognition, and stronger perceived emotionality (e.g., Bourne, 2010). Furthermore, right hemisphere deficiencies have been shown to relate to difficulties in emotional facial expression recognition, as well as with difficulties in general social and emotional functioning (e.g., Meletti et al., 2003); Murray et al., 2015). However, other studies show a more varied picture, providing evidence for both the right hemisphere as well as the valence hypothesis (e.g., Wyczesany, Capotosto, Zappasodi, & Prete, 2018). For example, a recent study in which behavioral, that is, as well as electrophysiological data were collected of participants while they viewed faces presented in either the left or right visual field, or in both, the behavioral data were more in support of the valence hypothesis, while the electrophysiological data were more in line with the right hemisphere hypothesis (Prete, Capotosto, Zappasodi, & Tommasi, 2018). All in all, while the main evidence appears to suggests that the right hemisphere generally plays a more important role in emotion processing than the left hemisphere (Murray et al., 2015), evidence is definitely not conclusive and it is suggested that the two main hypotheses regarding the hemispheric lateralization of emotion processing are not mutually exclusive (Prete et al., 2018). Therefore, though this study is mainly focused on examining possible differences in lateralization of emotion processing between VS patients with and without facial paresis, we will examine the overall lateralization—in line with the right hemisphere hypothesis—as well as possible differences in lateralization based on valence—in line with the valence hypothesis.

The current's study addresses the lateralization of hemispheric processing by a method that has been extensively used in previous research: The chimeric faces test, a behavioral test of facial emotion processing which presents a face with an emotional expression in one half of the face and a neutral expression in the other half of the face. The image of the face is presented centrally, with the emotional facial expression thus being presented either in the left or the right visual field. This test examines whether there exists a bias in the observer considering the perception of emotional expressions presented in the left compared to the right visual field (e.g., Bourne, 2010; Bourne & Gray, 2011; Levy, Heller, Banich, & Burton, 1983). Hemispheric lateralization of emotion processing concerns the bias people tend to show in perceiving emotional expressions shown in the left or the right visual field as more emotional, or to recognize them more accurately depending on the visual field in which they are portrayed (Bourne, 2010; Murray et al., 2015). Considering that the information that is shown in the left visual field initially is received and processed by the right brain hemisphere, a left visual field bias is interpreted as support for the notion that the right hemisphere is more strongly involved in emotion processing than the left hemisphere (Bourne, 2006).

The role of the facial muscles of the observer in relation to hemispheric lateralization has been partly examined in healthy individuals as well as patients with mild unilateral facial paralysis (Blom, Aarts, & Semin, 2019; Korb et al., 2016) First, a recent study (Blom et al., 2019) using the chimeric faces test reported typical left visual field bias on perceived emotionality, but this visual field bias did not directly emerge in facial muscle activation. Furthermore, a study testing patients with acute, subacute or chronic unilateral facial paresis found that patients with a left versus right facial paresis processed emotional expression of happiness and anger equally. Interestingly, patients with a left facial paresis processed happy expressions more accurately when presented in the right versus left visual field, indicating a somewhat complicated relationship between facial paresis and emotional processing of others’ expressions (Korb et al., 2016). In short, although suggestive, the research conducted so far does not give a clear picture about the role of facial muscles in perceiving emotionality in facial expressions of others.

The current study aims to enhance the understanding of the possible role of facial mimicry in perceived emotionality by examining the impact of being limited in one's facial functioning on emotion processing of hemispheric lateralization. First of all, while the left visual field bias—in line with the right hemisphere hypothesis—has often been observed in healthy individuals, we aim to replicate this typical bias effect in a sample of patients with VS. Additionally, we test for possible differences in bias based on the valence of the emotional expression. If the patients show a left versus right visual field bias for positive versus negative facial expressions, this would relate to the valence hypothesis. Most importantly, however, we examined the role of facial functioning in hemispheric lateralization of emotion processing by comparing VS patients with and without facial paresis, as well as by examining the association between hemispheric lateralization of emotion processing and the degree of facial dysfunction as measured by the House Brackmann Grading scale (HBG; House, 1985). If facial functioning plays an important role in this, patients’ facial functioning should be related to the visual field bias.

## 2 MATERIAL AND METHODS

### 2.1 Study overview

We investigated the role of facial functioning in how emotional patients with VS perceive faces showing emotional expressions in the left or right visual field, with the other visual field being neutral in expression. Treatment of VS can include surgical removal of the tumor that causes a degree of (chronic) unilateral paresis in the patient. To take this important facial functioning difference into account, the study had a mixed design with emotional expression (angry vs. happy) and emotional half (left vs. right visual field) of the stimulus as repeated measures, with facial functioning (patients with or without facial paresis) as the main independent variable. The study was conducted and written informed consent of each participant was obtained in compliance with the principles contained in the Declaration of Helsinki. Permission for the study was granted by the Medical Ethics Committee of the Leiden University Medical Center.

### 2.2 Participants

Incidence rate of VS is low, with an estimated incidence rate of 15 persons per million in the Netherlands—where the current study took place—, with the highest latest incidence rate in one specific region of the Netherlands being 33.2 (Kleijwegt, Ho, Visser, Godefroy, & van der Mey, 2016). Clearly, the number of VS patients experiencing a chronic condition of facial paresis due to surgical removal of the VS is even much lower. Considering this low incidence rate, we aimed at including a reasonable number of VS patients with or without facial paresis (*N* = 44) to examine interaction effects within our mixed design with two within subject repeated measures. Running a sensitivity analysis in G*Power 3.1 (*α* = 0.05, power = 80%, *N* = 44) for an ANOVA: Repeated measures within–between interaction (including the moderator test of the patient group as well) indicated that we were able to detect a small to moderate effect size, *f* = 0.18.

All patients participating in the current experiment had previously been diagnosed with VS in the Leiden University Medical Center and the Radboud University Medical Center Nijmegen. In order to obtain a clear view of the specific impact of a unilateral facial paresis on lateralization of emotion processing, we aimed for a patient sample of which half had a chronic condition of unilateral facial paresis, while the other half of the sample had not developed a facial paresis at all.

Patients with and without facial paresis were matched as closely as possible (see Table 1 for details of the two subsamples) on the factors biological sex, age, side of the VS and the time that had elapsed since their diagnosis. In total, 28 females and 16 males participated (*M*_age_ = 54.39 years, *SD* = 7.41 years). Twenty‐two patients experienced a degree of facial paresis after removal of their VS, while twenty‐two patients had a VS but had not developed a facial paresis. Seventeen patients had a VS in the right cerebello pontine angle, while twenty‐seven patients had it in the left cerebello pontine angle. The average time that had passed since being diagnosed with VS was 6.55 years (*SD* = 4.74). Facial dysfunction was graded by means of the House Brackman Grading scale (HBG); currently, the most commonly used and accepted scale to document patients’ degree of facial dysfunction (Zandian et al., 2014). This scale contains six levels of facial nerve function, with a higher grade representing stronger facial dysfunction. The HBG was scored both by the experimenter and by the patients themselves. Inter‐rater reliability was high: Pearson's *r* = .87, therefore, the average HBG was used for analyses.

**TABLE 1:** Descriptives of VS patients with and without facial paresis

	Patients without facial paresis	Patients with facial paresis
Age in years	*M* = 55.32, *SD* = 6.99	*M* = 53.45, *SD* = 7.85
Sex	Female (14), Male (8)	Female (14), Male (8)
Handedness	Left (1), Right (18), Mixed (0)	Left (2), Right (19), Mixed (1)
Average HBG	*M* = 1.28, *SD* = 0.56	*M = *3.85, *SD* = 1.15
Localization VS	Left CPA (14), Right CPA (8)	Left CPA (13), Right CPA (9)
Time since diagnosis in years	*M = *5.92, *SD* = 3.84	*M = *7.19, *SD* = 5.51

Note

The number of patients in each category is reported between brackets when applicable. Of the patients without facial paresis, we lack the information on handedness of three individuals.

Abbreviations: CPA, Cerebellopontine angle; HBG, House Brackman Grade.

#### 2.2.1 Participant recruitment and response rate

Patients applied for participation either via responding to a letter of invitation received from their treating physician, or via responding to a call for participants on an online forum for people with VS.^1^The Dutch website for vestibular schwannomas: www.brughoektumor.nl. Out of the 62 patients who applied either via the online forum or who were invited by their treating physician, 42 (70.79%) decided to participate in the current experiment.

### 2.3 Stimuli

**FIGURE 1:**
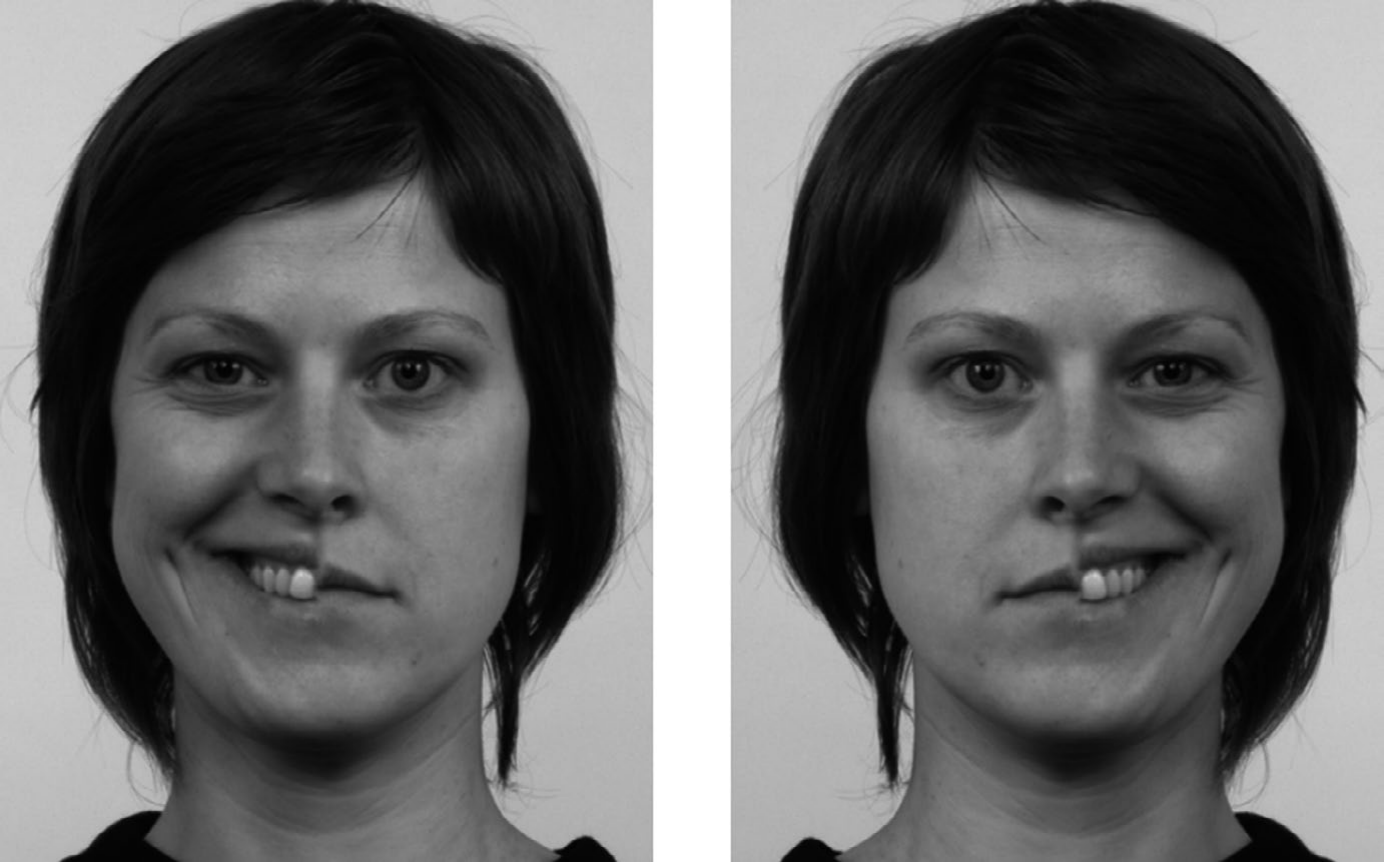
Examples of a chimeric face showing a happy facial expression in the left visual field (left image) and in the right visual field (right image)

Chimeric faces that were created for and used in an earlier study (Blom et al., 2019) were used in this study as well. The chimeric faces were generated using images of four female and four male faces from the Dutch Radboud Faces Database (Langner et al., 2010). Each chimeric face was composed of an emotional (angry or happy) half face and a neutral half face (see Figure 1 for an example) of the same model, by blending the faces at the midline. We used both the original pictures and the mirrored pictures. The effects that we would find would then thus be due to a true visual field bias, not to a possible difference in expressiveness of the left or right side of the face of the poser. The final stimulus set consisted of 64 unique chimeric faces, differing in biological sex (4 male, 4 female), emotional expression (happy vs. angry), emotional visual field (left vs. right), and version (original vs. mirrored). The images of faces had a resolution of 462 × 562 pixels and an absolute size of 11.3 × 15.0 cm, and were presented in grayscale on a gray background.^2^These chimeric face stimuli are available upon request from author SB. The visual angle was not measured because participants’ head position was not fixed for the current experiment. Participants adjusted the distance to the laptop screen to their convenience.

### 2.4 Procedure

Patients were informed that they had to rate on a 9‐point scale—using the numeric keys 1 to 9—how emotional they found each face presented to them on the screen. They used their preferred hand to give their response and were asked to not think too long about their rating and to trust their first impression. After four practice trials, in which patients could get accustomed to the task and to the type of images, the experiment started. The task was presented in two blocks, each block consisting of the same 64 trials, presented randomly without replacement. Each trial started with a blank screen (1,000 ms), after which a fixation point appeared (random time between 600 and 1,000 ms). Then, the chimeric face appeared with the rating scale below the face, which remained on screen until the face was rated. Patients went through the experiment self‐paced and could take a break in between blocks if they felt the need to. Average ratings of emotionality were calculated per stimulus type and served as dependent variable.

### 2.5 Statistical analyses

We will first test the hypothesis that VS patients with and without facial paresis show differences in lateralization of facial emotion processing as measured by the visual field bias with classical statistical tests in the form of a mixed ANOVA (see Section 3.1). Normality of the data was confirmed by use of *Q*–*Q* plots as well the Kolmogorov–Smirnov test, and homogeneity of variances was confirmed by means of Levene's test for equality of variances. The sphericity assumption was met considering that each factor only consisted of two levels. Considering earlier research suggesting that the side of facial paresis matters for emotional processing of facial expressions (Korb et al., 2016), we include side of VS as an exploratory factor.

Next, we will perform a regression analysis with patients’ HBG as predictor in order to provide a more thorough view of the relationship between the degree of facial dysfunction in VS patients and their visual field bias (see Section 3.2). Before running this analysis, linearity was inspected by means of scatterplots, and homoscedasticity and normality was checked by use of *Q*–*Q* plots of the regression standardized residuals, the Kolmogorov–Smirnov test, and by means of normal *P*–*P* plots of the regression standardized residuals.

Lastly, we will compare the visual field bias of VS patients to the visual field bias of a healthy control sample (reported in Blom et al., 2019) by running an ANOVA (see Section 3.3). Homogeneity of variances was checked by means of Levene's test for equality of variances, and normality of the data was inspected by use of *Q*–*Q* plots as well the Kolmogorov–Smirnov test.

In addition to the classical statistical tests, Bayesian analyses are performed to quantify the evidence of the hypotheses under investigation. Bayesian Factors (BF) are reported, with a larger BF representing more evidence in the data set for the hypothesis under consideration.

## 3 RESULTS

### 3.1 Visual field bias and facial paresis in VS patients

In order to examine how patients respond to the chimeric faces, we performed an analysis of variance of the emotionality ratings as a function of emotional half (left vs. right visual field) and emotional expression (happy vs. angry) as within subject factors and facial paresis (present vs. absent) and side of VS (left vs. right Cerebellopontine angle, CPA) as between subject factors. The side of facial paresis was included as an exploratory factor, and it should be taken into account that the division of patients based on side of VS was not equal, given that 27 patients had their VS in the left, and only 17 had it in their right CPA. For the sake of clarity of reading, below we first report the main effects, followed by all higher order interaction effects.

#### 3.1.1 Main effects

First of all, a large main effect of emotional half showed, *F*(1, 40) = 28.06, *p* < .001, *η*_p_^2^ = 0.41. As expected, patients rated faces showing an emotional expression in the left visual field as more emotional (*M* = 6.47, *SD* = 0.96) than those showing an emotional expression in the right visual field (*M* = 6.07, *SD* = 1.03), mean difference 0.40, 95% CI [0.25, 0.54]. A Bayesian one sample *t*‐test revealed that the data were 15,532 times more likely to reflect a left visual field bias (BF_10_ = 15,532), than for it to reflect a null effect. The basic left visual field bias of the chimeric faces test was thus replicated with the current patient sample, by revealing a large effect of emotional half of the face.

Second, a main effect of emotional expression, *F*(1, 40) = 5.54, *p* = .024, *η*_p_^2^ = 0.12, showed that chimeric faces showing a happy emotional expression were rated as more emotional (*M* = 6.50, *SD* = 1.10) than those showing an angry emotional expression (*M* = 6.04, *SD* = 1.19), mean difference 0.46, 95% CI [0.09, 0.83]. A Bayesian one sample *t*‐test revealed that the data were 2.58 times more likely to reflect this difference in emotionality ratings based on the emotional expression (BF_10_ = 2.58), than for it to reflect a null effect.

No main effect of facial paresis emerged. VS patients with a facial paresis did not show overall differences in their emotionality ratings (*M* = 6.18, *SD* = 1.16) compared to VS patients without a facial paresis (*M* = 6.36, *SD* = 0.76), *F*(1, 40) = 0.15, *p* = .701, *η*_p_^2^ = 0.00. A Bayesian independent samples *t*‐test revealed that the data were 2.88 times more likely to reflect a null effect (BF_01_ = 2.88), than for it to reflect a difference in emotionality ratings based on facial paresis being present or absent. Also, the analysis did not yield a main effect of side of side of paresis. Patients who had the VS on the left side (*M* = 6.31, *SD* = 0.87) versus the right side (*M* = 6.21, *SD* = 1.14), did not show an overall difference in emotionality ratings, *F*(1, 40) = 0.09, *p* = .771, *η*_p_^2^ = 0.00. A Bayesian independent samples *t*‐test revealed that the data were 3.17 times more likely to reflect a null effect (BF_01_ = 3.17), than for it to reflect a difference in emotionality ratings based on the side of the facial paresis.

### 3.2 Two‐way interaction effects

The interaction between emotional half and valence of the emotional expression being positive or negative (happy vs. angry chimeric faces) showed to be significant, *F*(1, 40) = 4.43, *p* = .041, *η*_p_^2^ = 0.10. A larger difference based on visual field in which the emotion was portrayed showed for happy (*M*_difference_ = 0.50, *SD* = 0.72) compared to angry (*M*_difference_ = 0.29, *SD* = 0.34) chimeric faces. A Bayesian paired samples *t*‐test, however, revealed that the data were only 1.23 times more likely to reflect this difference in visual field bias based on emotional expression (BF_10_ = 1.23), than for it to reflect a null effect.

**FIGURE 2:**
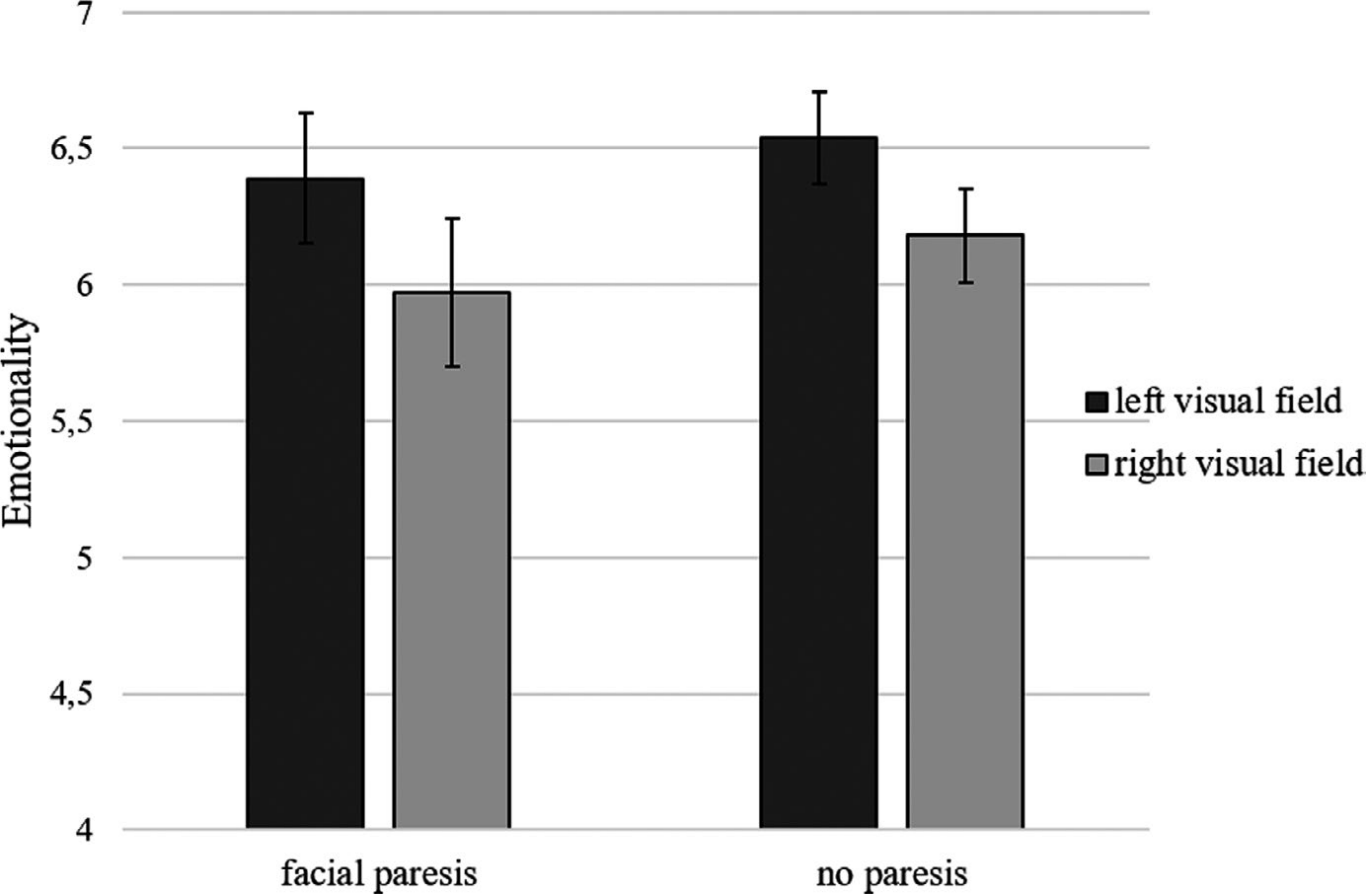
Left visual field bias in Vestibular Schwannoma patients. Error bars represent standard error

Furthermore, and important to the present hypothesis, the effect of emotional half was not qualified by an interaction with facial paresis, *F*(1, 40) = 0.15, *p* = .705, *η*_p_^2^ = 0.00, see Figure 2. Higher emotionality rating for emotional expressions shown in the left compared to the right visual field showed for VS patients without a facial paresis (*M*_difference_ = 0.37, *SD* = 0.41) as well as for VS patients with a facial paresis (*M*_difference_ = 0.42, *SD* = 0.52). A Bayesian independent samples *t*‐test revealed that the data were 3.16 times more likely to reflect a null effect (BF_01_ = 3.16), than for it to reflect a difference in overall visual field bias based on facial paresis being present or absent. The presence of a facial paresis thus most likely did not affect the visual field bias.

The analysis did not yield an interaction between emotional expression and facial paresis, *F*(1, 40) = 0.56, *p* = .457, *η*_p_^2^ = 0.01. Lastly, the exploratory factor side of VS did not show to interact with any of the other factors. No interaction showed between side of VS and facial paresis (present vs. absent), *F*(1, 40) = 0.74, *p* = .395, *η*_p_^2^ = 0.02, nor between side of VS and emotional expression (happy vs. angry), *F*(1, 40) = 0.18, *p* = .671, *η*_p_^2^ = 0.01, or between side of VS and emotional half (left vs. right visual field), *F*(1, 40) = 0.08, *p* = .773, *η*_p_^2^ = 0.00.

In line with this, a Bayesian analysis of variance indicated that neither the model including the interaction between emotional expression and facial paresis (BF_incl_ = 0.32), nor the model including the interaction between side of VS and paresis (BF_incl_ = 0.57), nor the model including the interaction between side of VS and emotional expression (BF_incl_ = 0.27), or the model including the interaction between side of VS and emotional half (BF_incl_ = 0.23) explained the data well compared to matched models not including these effects.

#### 3.2.1 Three‐way interaction effects

The interaction between emotional half and valence did not show to be qualified by a further interaction with facial paresis, *F*(1, 40) = 0.15, *p* = .704, *η*_p_^2^ = 0.00. A Bayesian independent samples *t*‐test revealed that the data were 3.03 times more likely to reflect this null effect (BF_01_ = 3.03), than for it to reflect a difference in visual field bias based on valence between the two patient groups.

Furthermore, no three‐way interaction showed between emotional expression, facial paresis, and side of VS, *F*(1, 40) = 0.50, *p* = .398, *η*_p_^2^ = 0.02, nor between emotional half, facial paresis, and side of VS, *F*(1, 40) = 0.34, *p* = .562, *η*_p_^2^ = 0.01. The Bayesian analysis of variance indicated that neither the model including the interaction between emotional expression, facial paresis, and side of VS (BF_incl_ = 0.50), nor the model including the interaction between emotional half, facial paresis, and side of VS (BF_incl_ = 0.32) explained the data well compared to matched models not including these effects.

#### 3.2.2 Four‐way interaction effect

Lastly, the interaction between emotional half, valence, facial paresis, and side of VS was not significant, *F*(1, 40) = 0.06, *p* = .806, *η*_p_^2^ = 0.00. The Bayesian analysis of variance indicated that the model including this interaction did not explain the data well compared to matched models not including this effect (BF_incl_ = 0.39).

To conclude, while the classic left visual field bias showed for the current patient sample, VS patients with and without facial paresis did not show a difference in this visual field bias (Figure 2). Furthermore, this left visual field bias showed to be slightly larger for happy than for angry chimeric faces.

### 3.3 Visual field bias and degree of facial dysfunction in VS patients

Second, it was examined whether the degree of facial dysfunction as measured by the average HBG score showed to be related to the above reported visual field bias. A score representing the visual field bias was computed by subtracting the average emotionality rating for faces with the emotional expression depicted in the right visual field, from those depicting the emotional expression in the left visual field. A positive visual field bias score thus represented a left visual field bias. A simple linear regression analysis with HBG score as independent variable and visual field bias score as dependent variable showed to be not significant, *F*(1, 42) = 0.08, *p* = .776, *R* ^2^ = .00. HBG thus did not predict the overall visual field bias b* = 0.04, *t*(42) = 0.29, *p* = .776, *B* = 0.01, 95% CI *B* [−0.08, 0.10]. A Bayesian correlation revealed that the data were indeed 5.11 times more likely to reflect a null effect (BF_01_ = 5.11), than for it to reflect an association between degree of facial dysfunction and the visual field bias.

We furthermore examined the relationship between the degree of facial dysfunction as measured by the average HBG score and the visual field bias for positive and negative (happy and angry chimeric faces). Separate scores representing the visual field bias for happy and angry chimeric faces were computed as described previously, with a positive visual field bias score again representing a left visual field bias.^3^Homoscedasticity was somewhat violated ‐as indicated by the Kolmogorov––Smirnov test‐ considering the dependent variable visual field bias of happy faces (*D*(44) = 0.154, *p* = .011) as well as the dependent variable visual field bias of negative faces (*D*(44) = 0.162, *p* = .005). For this reason, we applied a more conservative *p*‐value (.01 instead of .05) for the significance tests. It should be noted that this adjustment did not change our outcomes.


The association between HBG score and visual field bias score for happy chimeric faces was not significant, *F*(1, 42) = 0.00, *p* = .964, *R* ^2^ = .00. HBG thus did not predict the visual field bias for positive expressions b* = 0.04, *t*(42) = 0.05, *p* = .964, *B* = 0.00, 95% CI *B* [−0.14, 0.15]. A Bayesian correlation revealed that the data were indeed 5.32 times more likely to reflect a null effect (BF_01_ = 5.32), than for it to reflect an association between degree of facial dysfunction and the visual field bias for positive expressions.

The association between HBG score and visual field bias score for angry chimeric faces was also not significant, *F*(1, 42) = 0.46, *p* = .504, *R* ^2^ = .01. HBG thus did not predict the visual field bias for negative expressions b* = 0.10, *t*(42) = 0.67, *p* = .504, *B* = 0.02, 95% CI *B* [−0.05, 0.09]. A Bayesian linear regression revealed that the data were indeed 4.29 times more likely to reflect a null effect (BF_01_ = 4.29), than for it to reflect an association between degree of facial dysfunction and the visual field bias for negative expressions.

### 3.4 VS patient sample versus a healthy control sample

While we report a strong replication of the left visual field bias in the current patient sample, no relationship revealed between hemispheric lateralization of emotion processing and facial functioning of the current sample of patients with VS, neither with the mere presence or absence of a facial paresis nor with the degree of facial dysfunction (as measured by the HBG). The null effects regarding possible differences in lateralization of facial emotion processing based on VS patients’ facial paresis could first of all be due to the absence of such hypothesized effect of facial functioning. This would be in line with the results of a previous study showing no meaningful association between facial muscle activity in the form of facial mimicry and the visual field bias (Blom et al., 2019). On the other hand, the null effects could also be due to an affected visual field bias in the VS patient sample as a whole (i.e., irrespective of their facial functioning).

In order to test this, we compared the data of the current patient sample, to the data of a previous sample (*N* = 23) of healthy college students (Blom et al., 2019). Both studies made use of the exact same stimulus material as well as the same task and setup of the chimeric faces test. An analysis of variance with overall visual field bias as dependent variable, and group (VS patients without facial paresis, VS patients with facial paresis, and healthy controls) as between subject factor^4^Non‐normality revealed for the data for one of the three groups (VS patients with facial paresis, *D*(21) = 0.23, *p* = .007). We report one‐way ANOVA results, considering that is a robust test against the normality assumption. Inspecting the alternative non‐parametric one‐way ANOVA (the Kruskal‐Wallis test) suggests that the pattern of results does not change. No significant differences (*χ* ^2^ = 0.01, *p* = .995) were found among the three participant groups (VS patients without facial paresis, VS patients with facial paresis, healthy student sample). showed no significant effect of group, *F*(2, 64) = 0.04, *p* = .965, *η*_p_^2^ = 0.00. Bonferroni post hoc tests confirmed that there was no difference between the overall visual field bias of the healthy control sample (*M* = 0.38, *SD* = 0.46) and VS patients with facial paresis (*M* = 0.41, *SD* = 0.53), *p* = .997, nor between the healthy control sample and VS patients without facial paresis (*M* = 0.37, *SD* = 0.41), *p* = .980. A Bayesian ANOVA confirmed that the data were 7.82 times more likely to reflect a null effect (BF_01_ = 7.82), than for it to reflect a difference in visual field bias comparing healthy controls, VS patients with facial paresis, and VS patients without facial paresis. Accordingly, we consider it most likely that the null effects were due to the absence of a relationship between facial functioning and lateralization of facial emotion processing, and not because of an affected visual field bias in the VS patient sample as a whole.

## 4 DISCUSSION AND CONCLUSION

The current study was aimed at examining hemispheric lateralization of facial emotion processing by means of the chimeric faces test in Vestibular Schwannoma patients with and without facial paresis. First of all, we replicated the left visual field bias in this patient sample, meaning that when an emotional expression was depicted in the left visual field, rather than in the right visual field, the face was perceived as being more emotional. This left visual field bias showed to be somewhat stronger for positive (happy) than for negative (angry) facial expressions. Our findings are, therefore, in line with the right hemisphere hypothesis, and not with the valence hypothesis. No difference in this bias showed based on the mere presence or absence of a facial paresis, nor did it show to be associated with the specific degree of facial functioning of the patients. Furthermore, exploratory analyses revealed no relationship between the side of the facial paresis and the visual field bias. Lastly, no difference showed between the visual field bias of VS patients and a healthy control sample. All in all, VS patients with and without a facial paresis show the same type of hemispheric lateralization of facial emotion processing as has been reported in nonpatient samples and thus do not appear to differ in this facet of emotion processing.

The current findings suggest that facial functioning and facial mimicry are not vital for hemispheric lateralization of facial emotion processing. These findings are in line with previous research, showing no direct association between emotion processing of other's expressions and facial muscle activity in healthy participants (Blom et al., 2019). Another recent related study, however, reported that individuals with left facial paresis showed an opposite error pattern compared to individuals with a right facial paresis when detecting whether a happy facial expression first appeared in the left versus right visual field (Korb et al., 2016). There are some differences between the Korb et al. and our study that could explain this apparent disparity in findings. First, the present study examined and compared VS patients with and without a facial paresis (matched on various factors), while Korb et al. tested a varied group of patients with a facial paresis (including patients with acute—less than 6 weeks—to chronic—more than 4 months paresis). Second, we examined differences between patients with or without facial paresis in lateralization of perceived emotionality of facial expressions, while Korb et al. (2016) tested whether patients with a left or right facial paresis were able to detect in which visual field a happy facial expression first appeared. Though speculative, then, differences in patient groups and task measurements might have produced different findings between the studies as a result of tapping into different aspects of emotion processing (e.g., detection of emotion in faces vs. perceiving emotionality in faces).

Considering that the current study does provide a strong replication of the left visual field bias, a finding in line with numerous previous studies showing the occurrence of this hemispheric bias in facial emotion perception, and the absence of facial paresis effects suggests that processes other than facial mimicry play a more important role here.

First of all, the perceived emotional intensity of emotional facial expressions could involve a neural network that is distinctive from mimicking the emotional expression itself. Perceived intensity of emotion has been associated with a network implicating more rudimentary subcortical processing and related to activity of the amygdala and nucleus accumbens (e.g., Gainotti, 2012; Phan et al., 2004), whereas the act of mimicking facial expressions involves more cortical processing related to motor simulation of facial expressions, the posterior cingulate cortex, and medial temporal lobe structures (Schilbach, Eickhoff, Mojzisch, & Vogeley, 2008). Accordingly, a possible explanation for the current findings might be that encoding the emotionality of another person's facial expression might occur (partly) independent from the mere mimicry of the facial expression itself. Furthermore, a recent study showed that the recognition of facial expressions can be achieved via two routes, namely by relying mainly on visual information and by sensorimotor information such as facial mimicry (de la Rosa, Fademrecht, Bülthoff, Giese, & Curio, 2018). Extrapolating those findings to the current study would suggest that hemispheric lateralization of facial emotion processing might be a process that relies more on visual and subcortical information processing, rather than on sensorimotor information processing involved in simulating the facial expressions of others.

Though our findings could be interpreted as evidence against the role of facial mimicry in emotion processing, we would like to stress here that the findings reported in this study do not necessarily go against the important function of facial mimicry. Other information—such as the visual (de la Rosa et al., 2018)—can sometimes provide sufficient input in order to complete emotion processing tasks, hence reducing the “need” for facial mimicry for certain tasks (e.g., Arnold & Winkielman, 2019). For example, while facial mimicry did show to relate to the valence of the chimeric faces in a previous study (Blom et al., 2019), it did not show to relate to the visual field in which the expression was shown. Hence, though the facial muscles might react to the facial expressions shown in the paradigm used in the present study, participants apparently can judge the emotionality of presented faces without relying on the sensorimotor route. Relatedly, the task utilized by Korb et al. (2016) might have relied more on the sensorimotor route than the current studies’ task, hence providing a different account for their reported findings somewhat diverging from our present findings.

In closing, although the present study mainly aimed to address the role of facial functioning in emotional processing of facial expressions, we would like to stress that is of equal importance to study different facets of emotion processing in patients with a facial paresis, as well as in patients with cerebellar damage. Other studies have for example reported differences in emotion perception and regulation in individuals with cerebellar damage (e.g., Houston et al., 2018). We, therefore, believe that future studies could examine this further by use of additional tasks that have previously been proven insightful for individuals with facial paresis and/or cerebellar damage. We wish to note here that the current study is part of a larger project that examined possible differences in *emotion processing* of facial expressions as well as perceived quality of life, social function, and emotion between VS patients with and without facial paresis. This project aims to provide a first step in obtaining a more complete picture of emotion processing and emotion regulation in patients by using several experimental tasks as well as questionnaires (see Blom, Aarts, Wever, Kunst, & Semin, 2020; Blom, Aarts, Kunst, Wever & Semin, manuscript under review).

The current study is one of the few experimental studies on facial emotion processing in patients with a facial paresis, and patients with a VS in particular. Knowledge on emotion processes that are and that are not affected in VS patients’ with and without facial paresis informs health practitioners regarding the care they could provide patients with respect to their wellbeing. Although the present study suggests that facial paresis is not associated with impaired lateralization of emotion processing, future studies could focus on other types of facial emotion processing to further the understanding of the possible impact of a facial paresis on emotion processing.

## CONFLICT OF INTEREST

The authors report no conflict of interest.

## AUTHOR CONTRIBUTION

SB and GS designed the study. SB programmed the study, collected the data, prepared, and analyzed the data. SB wrote the manuscript in consultation with HA and GS. CW and HK helped in access to participants, and provided consultation on the parts of the manuscript about the VS patient sample specifically.

## Data Availability

A data set is available and stored and can be requested from the corresponding author.
